# Commentary: Case report: Hereditary fibrosing poikiloderma with tendon contractures, myopathy, and pulmonary fibrosis (POIKTMP) presenting with liver cirrhosis and steroid-responsive interstitial pneumonia

**DOI:** 10.3389/fgene.2023.1255807

**Published:** 2023-12-22

**Authors:** Filippo M. Panfili, Andrea Pietrobattista, Davide Vecchio, Michaela V. Gonfiantini, Andrea Bartuli, Marina Macchiaiolo

**Affiliations:** ^1^ Rare Diseases and Medical Genetics Unit, Bambino Gesù Children’s Hospital, IRCSS, Rome, Italy; ^2^ Division of Gastroenterology, Hepatology and Nutrition, Bambino Gesù Children’s Hospital IRCCS, Rome, Italy

**Keywords:** POIKTMP, FAM111B, POIK-TMPL, liver, cirrhosis, telomere, poikiloderma, fibrosis

## Introduction

A congenital multisystemic disorder known as POIKTMP (OMIM#615704) is caused by heterozygous *FAM111B* gene variants (OMIM#615704), which codes for FAM111B protease. This condition is mainly characterized by poikiloderma, tendon contractures, myopathy, and pulmonary fibrosis, with additional features including pancreatic exocrine insufficiency, cataract, pancreatic cancer, and liver involvement (Hoeger et al., 2023).

Takimoto-Sato et al., in their study, reported a new case of *FAM111B*-related disease in an adult patient, the first in Japan, characterized by all the classical clinical criteria, including severe pulmonary fibrosis and liver disease. The latter was diagnosed at the stage of cirrhosis with portal hypertension (PH) and hepatic encephalopathy, resulting in the cause of death (Takimoto-Sato et al., 2022).

## Liver involvement in *FAM111B*-related disease

Recently, we published a study describing a severe liver involvement in a 17-year-old patient with *FAM111B*-related disease, together with a literature review which allowed including 11 more patients previously described with variable degrees of liver impairment ranging from mild hepatomegaly to severe liver fibrosis with portal hypertension and even end-stage liver diseases (ESLDs) (Macchiaiolo et al., 2022). In this view, our study suggested expanding the POIKTMP phenotype to include liver disease formally and, thus, proposing the new acronym POIK-TMPL (POIKiloderma, Tendon contractures, Myopathy, Pulmonary fibrosis/Pancreatic insufficiency and cancer, Liver involvement/Lymphedema). Of regard, we were able to describe the natural history of the progressive liver disease in *FAM111B*-related disease, which ultimately resulted in being fatal, over a long follow-up of 17 years. In our patient, the hepatic impairment was present at diagnosis in the first years of life with an observation of cholestasis, hepatomegaly, and mild abnormal liver enzymes. Later, in the follow-up, the worsening of liver fibrosis and the development of PH became the more evident features with signs of protein synthetic dysfunction.

To date, the medical literature referred to a heterogeneous histologic pattern in *FAM111B*-related disease consisting of macrovesicular steatosis with few portal inflammatory cells, fibrosis which can cause disturbed architecture due to the porto-portal fibrous septa, possible loss of biliary ducts, and increased Kupffer cells, sometimes with a large leaf-like cytoplasm (Dokic et al., 2020; Macchiaiolo et al., 2022; Seo et al., 2016).

Particularly, hepato-pulmonary syndrome (HPS) should always be eliminated in patients with PH and impaired oxygenation using a reduced pulmonary function test, including DLCO. The presence of interstitial pneumonia has already been reported in association with HPS (Tercé G et al., 2010; Shahangian et al., 2014) and, thus, does not allow eliminating this severe condition.

In addition, corticosteroid treatment should be carefully evaluated in case of cirrhosis with some degree of decompensation because it can increase a protein load via increasing the protein turnover (Auron and Brophy, 2012). As a result, in this setting, steroids might provoke hyperammonemia, leading to worsening encephalopathy.

However, even if it was not the main aim of their study, the case reported by Takimoto et al. lacks extensive details on liver disease and its complications to further contribute to the topic of hepatic involvement in *FAM111B*-related disease.

In a recent paper, Arowolo et al. suggested a role of FAM111B protease in inadequate DNA repair, genome instability, chronic inflammation, aberrant apoptosis of the epithelial cells, and fibroblasts in different tissues, triggering a fibrosis progression (Arowolo et al., 2022; Hoeger et al., 2023).

Interestingly, in a recent paper, Kliszczak et al. highlighted how the abnormal FAM111B proteins in POIKTMP showed more prominent localization to the nuclear periphery, suggesting an interaction with the nuclear pore complex, and this was associated with an abnormal nuclear shape and an increase in micronuclei and ultra-fine DNA bridges that are hallmarks of genomic instability. Their findings demonstrated how, in the absence of FAM111B protease, there is a reduced recruitment of the shelterin component TRF2 that localizes at the telomere level, binding to core histones, to protect chromosome ends from inappropriate DNA damage response and loss of telomeric DNA, suggesting a possible role of TRF2 loss in telomere shortening in *FAM111B*-related disease (Konishi et al., 2016; Kliszczak et al., 2023). Indeed, in both telomerase-positive MCF7 cells and U2OS cells, which utilize alternative lengthening of telomeres (ALT), there is an increased level of telomere loss and fusion/intrachromosomal telomere signal events, independently of the other mechanisms of telomerase or recombination-driven telomere extension (Kliszczak et al., 2023). It is common knowledge that telomere shortening is linked to the progression of liver cirrhosis and that hepatic senescence markers are linked to disease progression and a poor prognosis (Carulli and Anzivino, 2014). Moreover, it is also known that rare constitutional missense variants in telomerase reverse transcriptase (TERT), the core enzyme for telomerase, are associated with a reduced telomerase activity and telomere size, leading to an increased risk of cirrhosis in adults. Rare cases of liver diseases have also been reported in patients with germline mutations in other genes involved in telomere biology, such as *TERC*, *DKC1*, and *RTEL1* (Nault et al., 2019). These findings suggest that telomerase mutations and, therefore, telomerase shortening may accelerate liver disease progression to cirrhosis in the context of chronic liver injury (Carulli and Anzivino, 2014).

## Discussion

We believe that these findings shed new light on the pathophysiology of this disease, unraveling a possible molecular mechanism of fibrosis not only of skin and lungs but also of the liver.

Further studies are needed to better evaluate the clinical course of liver involvement in this disease, leading to possible management of this complication, allowing to speculate on the potential use of several therapeutic strategies developed to target telomerase and ALT (Gao and Pickett, 2022). Until now, several immunosuppressant drugs have been used to treat the fibrotic involvements of the lungs, skin, and liver with scarce results (Dokic et al., 2020; Macchiaiolo et al., 2022; Takimoto-Sato et al., 2022).

Moreover, whether it is possible to hypothesize that exposure to toxic agents such as alcohol or hepatotoxic drugs should be avoided or reduced to minimize the effects of chronic liver injury remains unclear. Overall, the evidence of liver disease in FAM111B warrants a proper hepatology work-up at diagnosis and a dedicated follow-up, accounting for the known complications of cirrhosis in adults and children.
